# Pore-scale dynamics of enzyme adsorption, swelling and reactive dissolution determine sugar yield in hemicellulose hydrolysis for biofuel production

**DOI:** 10.1038/srep38173

**Published:** 2016-12-01

**Authors:** Sajal Kanti Dutta, Saikat Chakraborty

**Affiliations:** 1 Department of Chemical Engineering, Indian Institute of Technology, Kharagpur 721302, India; 2 School of Energy Science and Engineering, Indian Institute of Technology, Kharagpur 721302, India

## Abstract

Hemicelluloses are the earth’s second most abundant structural polymers, found in lignocellulosic biomass. Efficient enzymatic depolymerization of xylans by cleaving their β-(1 → 4)-glycosidic bonds to produce soluble sugars is instrumental to the cost-effective production of liquid biofuels. Here we show that the multi-scale two-phase process of enzymatic hydrolysis of amorphous hemicelluloses is dominated by its smallest scale–the pores. In the crucial first five hours, two to fourfold swelling of the xylan particles allow the enzymes to enter the pores and undergo rapid non-equilibrium adsorption on the pore surface before they hydrolyze the solid polymers, albeit non-competitively inhibited by the products xylose and xylobiose. Rapid pore-scale reactive dissolution increases the solid carbohydrate’s porosity to 80–90%. This tightly coupled experimental and theoretical study quantifies the complex temporal dynamics of the transport and reaction processes coupled across scales and phases to show that this unique pore-scale phenomenon can be exploited to accelerate the depolymerization of hemicelluloses to monomeric sugars in the first 5–6 h. We find that an ‘optimal substrate loading’ of 5 mg/ml (above which substrate inhibition sets in) accelerates non-equilibrium enzyme adsorption and solid hemicellulose depolymerization at the pore-scale, which contributes three-quarters of the soluble sugars produced for bio-alcohol fermentation.

The key to producing commercially viable, low carbon footprint lignocellulosic fuels is to supplement cellulosic ethanol with hemicellulosic alcohols^1^,^2^,^3^. Hemicelluloses–the earth’s second most abundant natural polymer after cellulose–constitute 10–50% of the biomass^4^,^5^, interlinking the cellulose chains inside the plant cell walls through hydrogen bonding^6^ and being covalently bonded to the lignin chains^7^. Cell wall deconstruction^8^ and hemicellulose depolymerization constitute the largest chunk of the resource pie spent on producing hemicellulosic fuels.

Hemicelluloses are structurally heterogeneous branched polysaccharides, composed of xylose monomer units linearly linked by *β*-(1 → 4)-glycosidic bonds, with galactose, glucose, manose, arabinose, 4-O-methylglucuronic acid, acetyl group, ferulic acid or p-cumaric acid as side chains. Thus, their depolymerization requires the synergistic effects of several enzymes such as the endoenzymes^9^ that cleave the *β*-(1 → 4)-glycosidic linear main chain randomly, the exo- and *β*-enzymes^9^,^10^ for the non-reducing ends, and the *α*-glucuronidase and *α*-L-arabinofuranosidase enzymes for the *α*-(1,2) and *α*-(1,3) covalent side chains^10^. A low Degree of Polymerization (DP), its branched structures and acetyl groups make the hemicellulose amorphous^11^,^12^, and easily accessible to the enzymes^13^.

Enzymatic hydrolysis of amorphous hemicelluloses is a multi-scale multi-step heterogeneous solid-liquid process involving the transport of soluble enzymes from the bulk liquid to the solid-liquid interface followed by pore diffusion, adsorption^14^,^15^,^16^ of their Carbohydrate Binding Domains (CBDs)^17^ to the insoluble substrate, formation of the enzyme-substrate complex and cleavage of the hemicellulose’s glycosidic bonds at the enzyme’s Catalytic Domains (CD) to produce smaller carbohydrate chains^18^, and the diffusive transport of the products (soluble sugars) out of the pores into the bulk phase^19^. These solid phase reactions are followed by liquid phase enzymatic hydrolysis of the soluble sugars to the monomers. The solid and liquid phase hydrolyses are both inhibited by the soluble products–the monomer (xylose) and the dimer (xylobiose)^20^,^21^. Other influencing factors include the surface morphology of the substrate^22^, interactions between the adsorbed enzymes^23^, surface diffusion of the adsorbed enzymes^24^, distribution of surface charges on substrates and enzymes^25^, steric hindrance by large groups in the side chains^10^, and reactor conditions such as temperature, pH, and ionic strength^26^,^27^. Adsorption equilibrium has been reported to attain in 2–4 h^27^,^28^,^29^, and hemicellulose hydrolysis has been simulated, using an equilibrium adsorption and lumped reaction kinetic model^30^, and a two-phase model with enzymatic reaction in the liquid phase alone^31^.

Most multi-scale reactive processes constitute of three representative scales, namely, the macro-scale (reactor), the meso-scale (pore), and the micro-scale (molecular), with the smallest (molecular) scale often being the most important determinant of the reaction rate.

A departure from this convention is observed when large carbohydrate molecules such as hemicelluloses are enzymatically hydrolyzed. For example, beechwood xylan–the hemicellulosic substrate primarily considered in this study–has a DP of 75–250^32^ with an average chain length of 170. Since the xylose monomer units (with Stokes diameter of 0.64 nm^33^) are linearly linked by β-(1 → 4)-glycosidic bonds, the molecular size of beechwood xylan would vary from 48–160 nm, averaging at 109 nm. Our pore size studies using Brunauer–Emmett–Teller (BET) and Barrett-Joyer-Halenda (BJH) methods (Supplementary Information, section I) show that the average pore size of beechwood xylan is 9.8 nm, which is tenfold smaller than its average molecular size, which in turn is significantly smaller than the reactor length scale. Thus, the sequence of length of scales participating in the multi-scale enzymatic hydrolysis of long-chain porous hemicelluloses such as beechwood/hardwood/softwood xylan, arabinoxylan, etc., is: *Reactor Scale* > *Molecular Scale* > *Pore Scale.*

This work is guided by the premise that the transport and reaction processes that determine product yields in enzymatic hydrolysis of amorphous natural polymers (such as xylan), where the pore-scale is the smallest of the three representative scales, are fundamentally different from other multi-scale catalytic reactions where the molecular scale is the smallest. We use a tightly coupled experimental and theoretical approach to quantify the complex temporal dynamics of the fundamental transport and reaction processes, coupled across scales and phases. We show that the secret to rapidly producing soluble sugars from amorphous natural polymers such as hemicelluloses lies in their smallest scale–the pores.

## Results and Discussion

**Figure 1:**
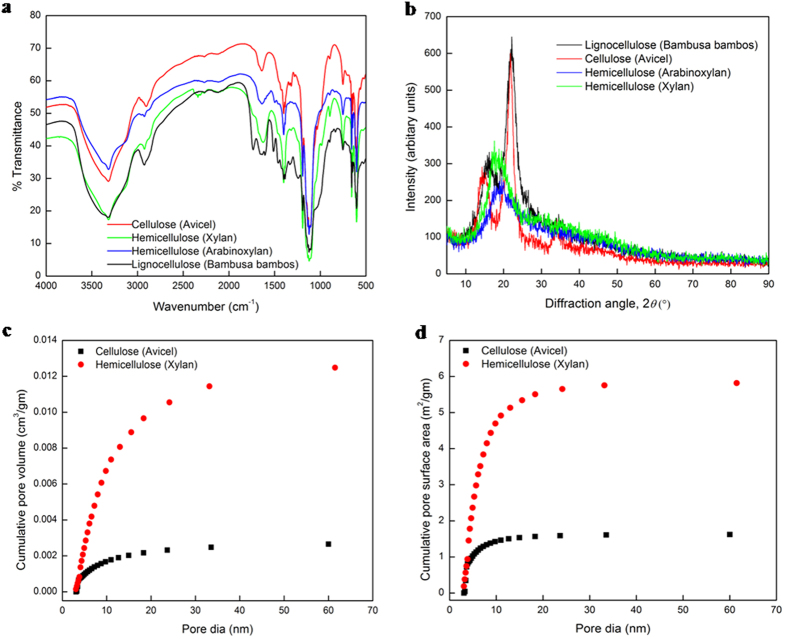
Characterization of cellulosic (Avicel), hemicellulosic (Xylan, Arabinoxylan) and lignocellulosic (*Bambusa bambos*) substrates. (**a**) FTIR spectra. (**b**) XRD patterns. (**c,d**) Pore size distribution derived from the desorption branch by the Barrett-Joyer-Halenda (BJH) method for cellulose and hemicellulose substrates with porosities of 0.42 and 5.73%, respectively.

We investigate if the dominance of pore-scale is unique to hemicelluloses alone by conducting FTIR, XRD, BET and BJH studies on four biomass substrates, namely, cellulose (Avicel PH101), hemicelluloses (arabinoxylan and beechwood xylan) and lignocellulose (*Bambusa bambos*; composition: 51.5% cellulose, 16.1% hemicellulose, 18.8% lignin). The FTIR spectra in transmittance mode show vibration bands representing similar bonds in all four natural polymers (Fig. 1a): O-H, C-H, C=C, H-O-H, C-C, C-O-C, C-O, along with the glycosidic bonds (Supplementary Information, section II).

The XRD patterns (Fig. 1b) show sharp peaks at 22.8° in the diffractogram for cellulose and *Bambusa bambos*, with relative crystallinities of 54.2% and 52.2%, respectively, (Supplementary Information, section II), while the broad peaks at 20° and negative values of crystallinity index (CI) for hemicelluloses (xylan, arabinoxylan) attest to their amorphous nature, with xylan shown to be more amorphous (i.e., more negative CI) than arabinoxylan.

The xylan substrate shows a porosity of 5.73%, an average pore size of 9.8 nm, a total external (BET) surface area of 5.27 m^2^/gm, and a total pore volume and total pore (BJH) surface area of 0.01332 cm^3^/gm and 5.837 m^2^/gm, respectively. The corresponding numbers for the cellulose (Avicel) are 0.42%, 4.09 nm, 1.01 m^2^/gm, 0.00284 cm^3^/gm and 1.622 m^2^/gm. While the pore size variation from 2 to 50 nm in both Avicel and xylan (Fig. 1b,c) confirms their mesoporous structures^34^, we note the significantly higher pore surface area (3.6X), pore size (2.4X), and pore volume (4.7X) in the hemicellulose than in the cellulose. Higher pore surface area accelerates the initial surface adsorption of enzymes while larger pore size and volume/higher porosity engender a larger reaction volume in the pores for hemicellulose hydrolysis. Furthermore, while 60% of the total pore volume and 88% of the total pore surface area of the cellulose result from pores smaller than 10 nm, the corresponding numbers for the hemicellulose are 50.5% and 80%, respectively (Fig. 1c,d).

**Figure 2:**
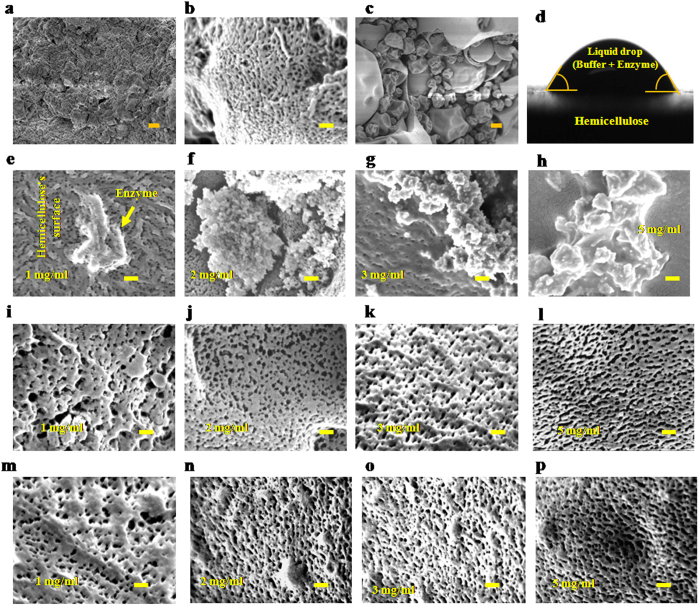
Surface morphology and contact angle. (**a–c,e–p**) FESEM images. (**a**) Beechwood xylan. (**b**) Pores on the surface of beechwood xylan. (**c**) Endoxylanase. (**d**) Contact angles between liquid and hemicellulose’s surface measured by Goniometer (ram´e-hart Germany) are 39.9 ° (left) and 40.6 ° (right). (**e**–**h**) Adsorbed enzyme on the solid hemicellulose at 4 h and *E*_0_/*C*_*s*_ = 0.1. (**i**–**l**) Pores structure on the surface of solid residue obtained from the enzymatic hydrolysis of xylan at 40 °C and at 1 h. (**m**–**p**) Pores structure on the solid surface at 5 h. In (**i**–**p**) the solid residues are more porous than in (**b**) due to the rapid pore-scale dissolution of the solid. Scale bars: (**a,c**) 1 μm; (**b,e**–**p**) 100 nm.

FESEM images (Fig. 2) provide visual proof of the xylan’s porous structures (Fig. 2a,b) amenable to adsorption and catalysis by the endoxylanase enzyme (Fig. 2c) dissolved in the buffer solution (Fig. 2d).

### Enzyme adsorption: equilibrium or non-equilibrium?

**Figure 3:**
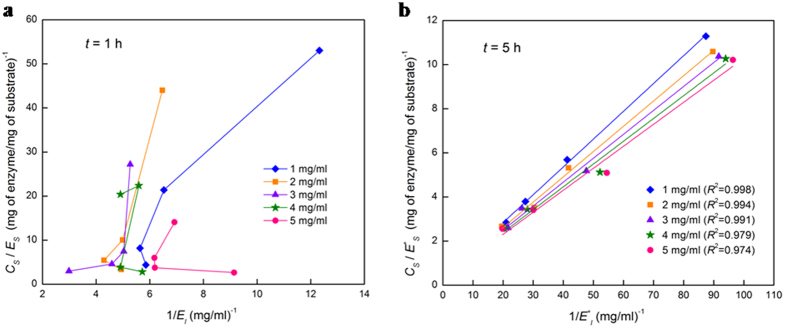
Nature of enzyme adsorption on the solid hemicellulose surface. (**a**) Non-equilibrium adsorption of enzyme at 1 h. (**b**) Equilibrium adsorption of the Langmuir-type at 5 h. Solid points are the (mean) experimental data (from runs in 0.1 M sodium acetate buffer solution of pH 5 at 4 °C, *C*_*s*_ = 1–5 mg/ml and *E*_0_/*C*_*s*_ = 0.1–0.4), and solid lines are the best-fits.

**Figure 4:**
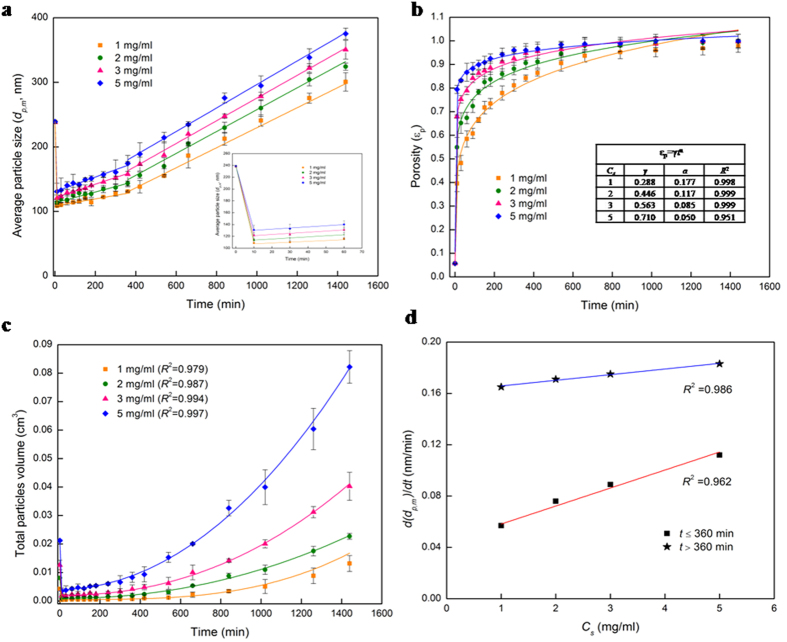
Surface and pore-scale reactive dissolution of xylan: porosity and swelling. (**a–c**) Temporal transformation of the physical properties of the solid substrate measured by Dynamic Light Scattering (DLS) techniques during the enzymatic hydrolysis of hemicellulose. Solid points are the experimental data and the solid lines are the best-fits. In (**a**) the change of average particle size indicates the initial rapid surface dissolution of the solid particles for 10 min (inset), followed by the swelling of the polymer particles for 11 h (i.e., the rest of the two-phase hydrolysis). In (**b**) 80–90% increase of porosity in the first 5 hours of hydrolysis suggests rapid pore-scale dissolution driven by the pore pressure drop. In (**c**) the degree of swelling of the polymers during hydrolysis is 2–4 for 1–5 mg/ml substrate loading. (**d**) The effect of the substrate loading on the *d(d*_*p,m*_)/*dt* (i.e., temporal rate of particle swelling) is a linear increase.

**Figure 5:**
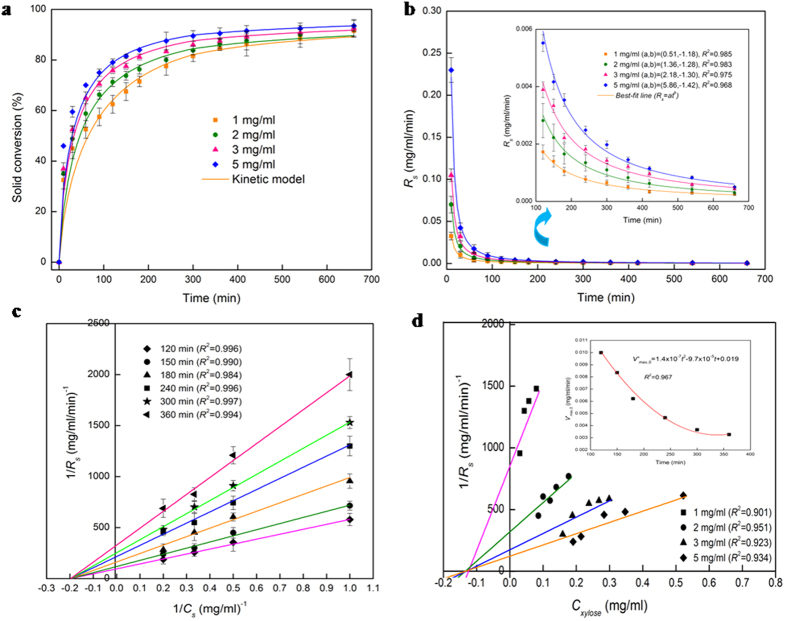
Solid hemicellulose conversion and solubilization rate, phase transition, and product inhibition. (**a**) Temporal dynamics of solid hemicellulose conversion in enzymatic hydrolysis. (**b**) Temporal variation of hemicellulose solubilization rate (*R*_*s*_) from the solid phase. (**c**) Non-competitive product inhibition in the solid phase. (**d**) Xylose inhibition in the solid phase. In (**a**) solid conversion (≥92%) at 11 h suggests the transition from two-phase (solid-liquid) hydrolysis to a single (liquid) phase one. Inset of (**d**) is the plot of apparent maximum velocity (*V′*_*max,s*_) versus time, and the maximum reaction rate (*V*_*max,s*_) is the extrapolated value of the fitted quadratic expression to *t* = 0. Solid points are the experimental results and the solid lines represent the simulation results (**a**) and the best-fit curves (**b,c,d**).

FESEM images (Fig. 2e–h) show an increase in enzyme adsorption as the xylan concentration increases gradually from 1 mg/ml (Fig. 2e) to 5 mg/ml (Fig. 2h), leading to faster reaction and rise in porosity at higher loading (Fig. 2l,p) than at lower (Fig. 2i,m). The temporal increase in porosity is also observed as the hydrolysis time increases from *t* = *0* (Fig. 2b) through *t* = 1 h (Fig. 2i,j,k,l) to *t* = 5 h (Fig. 2m,n,o,p) for various initial substrate loadings (1, 2, 3, 5 mg/ml).

This strong dependence of reaction rate on substrate loading allows us to hypothesize that the acetylated amorphous hemicellulose (Fig. 1a,b) initially promotes non-equilibrium adsorption of the endoxylanase, which does not follow equilibrium isotherms such as the Langmuir model^35^, given by


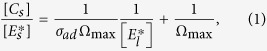


where *C*_*s*_ is the solid concentration (mg/ml), 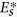 and 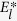 are the equilibrium concentrations of the free enzymes in the solid and liquid phases (mg/ml), respectively, *σ*_*ad*_ ( = *k*_*ads*_/*k*_*des*_) is the adsorption equilibrium constant (ml/mg), *k*_*ads*_ (ml/mg/min) and *k*_*des*_ (min^−1^) are the adsorption and desorption rate constants, respectively, and Ω_*max*_ is the maximum enzyme adsorption on the solid substrate (mg of enzyme/mg of substrate). To test our hypothesis, we perform adsorption experiments on the xylan-endoxylanase system for various initial substrate concentrations, and plot *C*_*s*_/*E*_*s*_ versus 1/*E*_*l*_ at different adsorption times to check if they follow the Langmuir isotherm (equation (1)). Two such representative plots show that the non-linear association between *C*_*s*_/*E*_*s*_ and 1/*E*_*l*_, indicating non-equilibrium adsorption at 1 h (Fig. 3a), and a best-fit linear association between *C*_*s*_/E^*^_s_ and 1/E^*^_l_, suggesting the onset of adsorption equilibrium at 5 h (Fig. 3b). The maximum enzyme adsorption (Ω_*max*_) and the adsorption equilibrium constant (*σ*_*ad*_) are calculated from the slope and the intercept (Fig. 3b) as 2.98–3.07 mg of enzyme/mg of substrate and 3.02–3.09 ml/mg, respectively, for substrate loadings of 1–5 mg/ml, suggesting that the equilibrium adsorption parameters (Ω_*max*_, *σ*_*ad*_) are practically independent of substrate loadings. The increase in the enzyme’s specific adsorption (*E*_*s*_/*C*_*s*_) with substrate loading (0.052–0.096 mg of enzyme/mg of substrate for 1–5 mg/ml loading at 4 h (Fig. 2e–h)) can be attributed to the positive charge on the endoxylanase (isoelectric point 9.0^36^) at pH 5^37^, while the xylan remains negatively charged^25^.

However, the question remains if this adsorption happens primarily on the external or the pore surface. Table 1 answers this by comparing the enzyme adsorption for the above (control) case with the enzyme adsorption on xylan particles soaked in the buffer solution for 5 h prior to the onset of enzyme adsorption. Porous hemicellulose particles under control conditions allow enzymes to adsorb both to the external and pore surfaces, while pre-soaked xylan particles with pores already filled with the buffer solution allow enzyme adsorption only on the external surface. The amount of enzymes adsorbed to the pore surface under control condition is significantly higher than that on external surface, both for non-equilibrium (at 1 h) and equilibrium (at 5 h) adsorption (Table 1), with the difference further increasing with xylan loading since higher substrate concentration (*C*_*S*_) for a constant total enzyme concentration (*E*_*0*_) provides greater relative availability of pore surfaces than external surfaces. As the adsorption progresses, the adsorbed enzyme concentration on the pores under control condition decreases as the adsorption time increases (1 h vs. 5 h), or as the total enzyme concentration increases for a constant substrate concentration.

### Pore-scale dissolution and hemicellulose swelling

A close look at Fig. 4a and its inset reveals that the average particle size (*d*_*p,m*_) of the substrate (measured by Dynamic Light Scattering (DLS)) decreases rapidly from 239 nm to 109–131 nm (depending on solid loading) in the first 10 min due to reaction-driven dissolution of the solid xylan. Then the amorphous xylan particles swell two to fourfold in volume (Fig. 4c), due to the capillary action resulting from the pore pressure drop^38^ (Δ*P*_*pore*_ = 4*σ*(cos*θ*)/*d*_*pore*_). This leads to monotonic temporal increase in the average particle size *d*_*p,m*_ (Fig. 4a) and the total particle volume *V*_*s*_ (Fig. 4c) (Supplementary Information, section III). Interestingly, the particles swell at a constant rate for the first 6 h, following which their swelling rate increases to a higher (loading-dependent) constant value (Fig. 4d).

The reversible enzyme adsorption-desorption is represented by


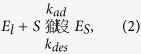


where *S* represents the available sites for adsorption of enzyme on the solid substrate (=[*Θ*_*max*_]*–*[*E*_*s*_])*, Θ*_*max*_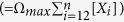 represents the maximum adsorption sites on the substrate, *E*_*s*_ is the adsorbed enzyme concentration, *E*_*l*_is the free liquid phase enzyme concentration, [*X*_*i*_] is the xylan concentration of chain length *i*, and 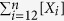 represents the total hemicellulose concentration in the solid phase at any time *t.* The rate of non-equilibrium adsorption of the enzyme on the solid surface is given by (Supplementary Information, section IV)


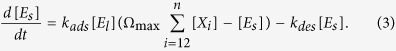


**Figure 6:**
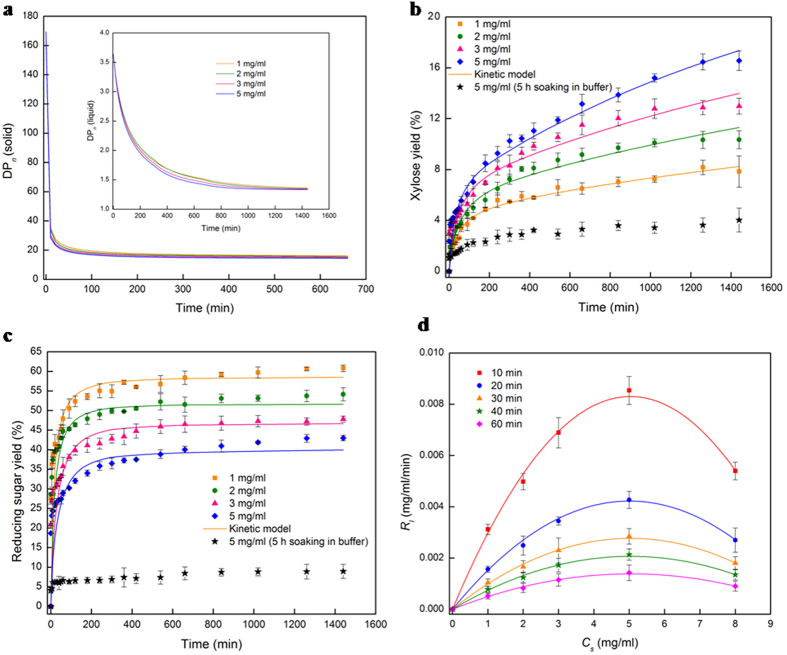
Number average Degree of Polymerization (DP_n_), sugar yields, and optimal substrate loading. (**a**) Temporal Dynamics of the number average Degree of Polymerization (DP_*n*_) in the solid phase, and inset of (**a**) is in the liquid phase in enzymatic hydrolysis of xylan. (**b**) Xylose yield. (**c**) Reducing sugar yield. (**d**) Substrate inhibition in the liquid phase. In (**b**) and (**c**) the yields of xylose and reducing sugar are obtained from the expression: *Y* = *C/C*_*s*_, where *Y* and *C* represent the yield and the concentration of the products in the liquid phase, respectively. In (**d**) the reaction rate in the liquid phase decreases with substrate loading above 5 mg/ml, and *C*_*s*_ = 5 mg/ml is obtained as the ‘optimal substrate loading’. Solid points are the experimental data, and solid lines are the model simulations (**a,b,c**) and the best-fit quadratic curves (**d**).

Our results, when viewed in the light of equation (3), begin to reveal the fundamental phenomena driving this multi-scale process of hemicelllulose hydrolysis. Equation (3) shows that more is the solid carbohydrate loading (
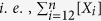
) available for non-equilibrium adsorption, faster is the enzyme adsorption, resulting in rapider solid hydrolysis to reducing sugars (*i* ≤ *11*) and reactive dissolution leading to higher porosity. Figure 4a (inset) allows us to infer that in the first 10 min, the non-equilibrium enzyme adsorption occurs more on the easily-accessible external surface of the hemicellulose particles, leading to rapid solid conversion of 32–46% (Fig. 5a) and a sudden decrease in particle size (from 239 nm to 109–131 nm) due to surface dissolution. The hydrolyzing media enters the pores of the xylan particles due to the pore pressure drop Δ*P*_*pore*_–driven capillary action (Supplementary Information, section III), and begins to swell the carbohydrate polymer particles when the latter’s percolation threshold is crossed after the first 10 min of hydrolysis. In addition to the capillary action, the Coulombic force of attraction between the enzyme molecules (which are positively charged in the buffer solution of pH 5^36^,^37^) and the negatively charged^25^ xylan particles further accelerates the transport of the xylanase molecules into the pores. Non-equilibrium enzyme adsorption continues on the pore surfaces (which initially offer 1.11 times more interfacial area than the external surface) for the first 5 h, leading to an increase in the solid phase conversion to 81–90% for 1–5 mg/ml (Fig. 5a), a power-law rise in porosity *ε*_*p*_(*t*) (Supplementary Information, section III) to 84–96% (Fig. 4b) due to pore-scale reactive dissolution, and the swelling of the particles from 109–131 nm to 126–161 nm (Fig. 4a). This non-equilibrium adsorption-driven rapid solid phase hydrolysis propels a sudden increase in the liquid phase yields of xylose (Fig. 6b) and reducing sugars (Fig. 6c) to 5.5–10.2% and 54.9–36.5%, respectively, for 1–5 mg/ml substrate loading, accompanied by a sharp decrease in DP_n_ (Fig. 6a). The soluble sugar yield plateaus out during the equilibrium phase of adsorption, increasing by a mere 5–6% after 5 h (Fig. 6c). Thus, the hydrolysis system, which is dominated by the surface reaction/molecular scale in the first 10 min, is governed by the swelling, non-equilibrium adsorption and reaction at the pore-scale for the first 5 h. Though the process continuous to be dominated by the pore-scale during the subsequent equilibrium adsorption, the rates of enzyme adsorption and carbohydrate depolymerization slow down drastically, with the conversion and porosity increasing by 4–10% and 2–10%, respectively, in the next 6 h. The deceleration of the reaction rate is accompanied by further swelling of the hemicellulose particles from 126–161 nm to 186–235 nm, which allows the xylose and xylobiose-rich hydrolyzing media to enter the pores and non-competitively inhibit the solid hydrolysis by binding to the free CBMs of the enzyme. When the porosity increases to 94–98% at 11 h, the hydrolysis is assumed to transition from a two-phase (solid-liquid) system to a single-phase (liquid) one.

Surprisingly, blocking the pore-scale adsorption by pre-soaking the particles for 5 h prior to the onset of enzymatic hydrolysis results in more than fourfold reduction in the sugar yields: for a 24 h hydrolysis with xylan loading of 5 mg/ml, the reducing sugar yield decreases from 43% (control) to 9% (Fig. 6c, bottommost curve) and the xylose yield decreases from 16.6% to 4% (Fig. 6b), suggesting that 79% of the solid hydrolysis occurs in the pores while the rest 21% happens from the external surface. The pre-soaked particles fail to provide the pore pressure drop (Δ*P*_*pore*_) needed by the enzymes to reach the pores through capillary action. Moreover, since the *Na*^+^ ions dissociated from the sodium acetate buffer solution have already entered the pore spaces of the negatively charged xylan substrate during the 5 h of soaking, there exists no further Coulombic force of attraction that can transport the enzyme to the pores. The absence of capillary and Coulombic forces prevents the adsorption of the enzyme to the pore surface (Table 1), and restricts the enzyme adsorption and hemicellulose hydrolysis to the external surface alone. This results in four to fivefold reduction in the sugar yields (Fig. 6a,b), suggesting that only less than a quarter of the soluble sugars and xylose depolymerized from the hemicellulose are produced from hydrolysis at the solid surface while more than three-quarters are products of pore-scale hydrolysis. The pore-scale’s role in accelerating the enzyme adsorption and the hemicellulose hydrolysis, and in enhancing the sugar yields thus cannot be overemphasized.

Thus, within the span of 24 h of hemicellulose hydrolysis, this multi-scale reaction system transitions through a spectrum of scales: from being governed by the molecular scale in the first 10 min to being governed by the pore-scale for the next 11 h and by the macro/reactor scale from 11–24 h. However, since the first 11 h constitute the most crucial phase of the hemicellulose hydrolysis, we conclude that the overall reducing sugar yield (Fig. 6c) is determined by the pore-scale dynamics of polymer swelling, non-equilibrium adsorption of enzymes and pore-scale reactive dissolution of the solid carbohydrate.

### Solid-phase hydrolysis kinetics with product inhibition

The kinetics of enzyme adsorption is represented by equation (3), while the multi-step kinetics for producing soluble carbohydrate molecules (DP ≤ 11), the so-called reducing sugars^39^, with non-competitive inhibition by the products (xylose (*X*_*1*_) and xylobiose (*X*_*2*_)) in the liquid and solid phases, are given by


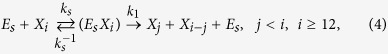



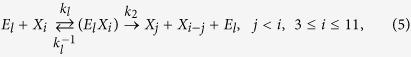



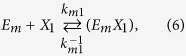



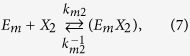



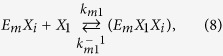



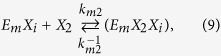



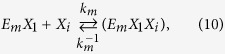



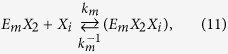


where *E*_*m*_ represents the free enzyme in the solid (*m* = *s*) or liquid (*m* = *l*) phase, *EX* and *EXX* with appropriate subscripts represent the enzyme-substrate and the enzyme-product complexes, *k*_*m*_, *k*^−1^_*m*_*, k*_*m1*_, *k*^−1^_*m*1_, *k*_*m*2_, and *k*^−1^_*m*2_ are the respective forward and backward reaction rate constants for the reversible reactions, and *k*_1_ and *k*_2_ are the reaction rate constants (min^−1^) for the irreversible reactions, respectively. The total amount of enzyme in the reactor is


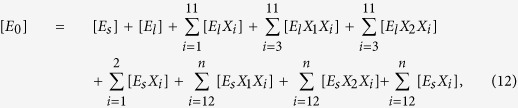


where [*E*_0_] is the initial enzyme concentration in the batch reactor. The temporal dynamics of the formation of reducing sugars (*i* ≤ 11) and low DP solid carbohydrates (*i* > 11) (Supplementary Information, section IV) are obtained by solving


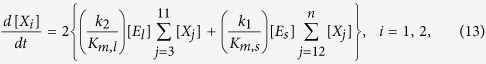



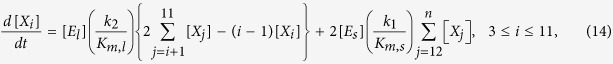



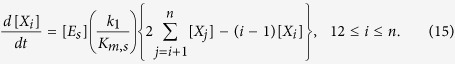


The products xylose and xylobiose inhibit the hydrolysis reaction by forming non-productive complexes in both the liquid and solid phases (equation (6)–(11)). We plot 1/*R*_*s*_ versus 1/*C*_*s*_ at various times to ascertain the type of product inhibition (non-competitive, competitive, mixed, or uncompetitive) in the solid phase hydrolysis. The equation for each best-fit straight line (Fig. 5c) is


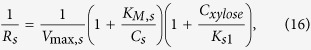


where *R*_*s*_, *V*_*max,s*_, *K*_*M,s*_, *C*_*s*_, *C*_*xylose*_, and *K*_*s*1_ are the solid depolymerization rate (mg/ml/min), maximum reaction velocity (mg/ml/min), Michaelis kinetic parameter (mg/ml), substrate concentration (mg/ml), xylose concentration (mg/ml), and xylose inhibition constant (mg/ml) in the solid phase, respectively. All the best-fit straight lines converge on the x-axis at −1/*K*_*M,s*_ (Fig. 5c) and the apparent maximum reaction rate (*V*′_*max,s*_) decreases with time (Fig. 5d (inset)), both showing that the product inhibition is non-competitive (*K*_*M,s*_ = 5.05 mg/ml; *V′*_*max,s*_ = 1.43 × 10^−7^*t*^2^−9.69 × 10^−7^*t* + 1.91 × 10^−2^, which when extrapolated to *t* = 0, gives *V*_*max,s*_ = 0.019 mg/ml/min). The point of intersection of all the best-fit straight lines (Fig. 5d, equation (16)) on the x-axis gives the solid phase xylose inhibition constant (*K*_*s*1_ = 0.132 mg/ml).

### Reducing sugar yield and substrate inhibition

The multi-step two-phase reaction kinetic model (equations (3) and (13, 14, 15)) is simulated (using NDSolve, Mathematica), with [*E*_*l*_] = [*E*_0_], [*E*_*s*_] = 0, and the normal distribution of DP_n_ of xylose polymers fractions in the substrate^32^ as the initial conditions at time *t* = 0, using liquid^40^ and solid phase kinetic data as well as adsorption data. The simulation results are validated against experimentally obtained temporal dynamics of solid conversion (Fig. 5a), and the xylose (Fig. 6b) and reducing sugar (Fig. 6c) yields in the liquid phase, at various substrate loadings. The non-equilibrium adsorption parameters^41^ (*k*_*ads*_, *k*_*des*_), and the xylobiose inhibition constants in the liquid (*K*_*l*2_) and solid (*K*_*s*2_) phases are determined through model-experiment comparison as *k*_*des*_ = *k*_*ads*_/*σ*_*ad*_, *K*_*l*2_ = δ*K*_*l*l_, *K*_*s*2_ = ξ*K*_*s*l_, where the model parameters are obtained as *k*_*ads*_ = 0.702 ml/mg/min, δ = 0.096, and ξ = 0.715 for all substrate loadings. The contrary trends in xylose and reducing sugar yields with increasing substrate loading (Fig. 6b,c) are attributed to the higher non-competitive product inhibition in the liquid phase than that in the solid (*K*_*s*1_ = 1.08*K*_*l*1_, *K*_*s*2_* *=* *8.06*K*_*l*2_). The formation of inhibitory complexes in the liquid phase prevents the hydrolyzed solids (Fig. 5a) from being completely available as reducing sugars (Fig. 6c).

*R*_*l*_ (liquid phase reaction rate) versus *C*_*s*_ (substrate loading) plots at various reaction times (Fig. 6d) show a biphasic response, with the liquid phase hydrolysis rate maximizing at a substrate loading of *C*_*s*_ = 5 mg/ml, which is the ‘optimal substrate loading’ (*C*_*s,max*_) for avoiding substrate inhibition (which has elsewhere^42^ been reported to happen at less than 1 mg/ml for xylan-xylanase system). The overall liquid phase hydrolysis reaction rate (*R*_*l*_) is given by


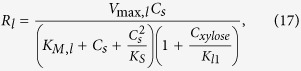


where *V*_*max,l*_ is the maximum liquid phase reaction velocity (mg/ml/min), *K*_*M,l*_ is the Michaelis kinetic parameter (mg/ml), and *K*_*S*_ is the substrate inhibition constant (mg/ml). Using equation (17), (d(*R*_*l*_)/*dC*_*s*_ = 0, at *C*_*s*_ = 5 mg/ml), we obtain *K*_*S*_ = 6.36 mg/ml, suggesting that the xylan-endoxylanse system is highly susceptible to substrate inhibition at high loading.

### Tracking timescales: which ones matter?

Table 2 shows that the various transport and reaction timescales associated with the process of enzymatic hydrolysis of hemicelluloses may be arranged as: *Pore scale diffusion time* < *Convective mixing time* < *Adsorption time* < *Desorption time* < *Liquid phase reaction time* < *Solid phase reaction time* < *Swelling time* < *Liquid phase diffusion time.* Simultaneous diffusion and convection transport the enzymes from the bulk to the solid surface, and convective mixing, being significantly faster than liquid phase diffusion (that has the largest timescale), determines the rate of enzyme and sugar transport in the liquid phase. The rest of the processes–pore diffusion, enzyme adsorption, desorption, solid phase reaction, particle swelling, liquid phase reaction–occur in series, and the timescale for reducing sugar production is a sum of these individual timescales (except that of the liquid phase reactions that produce xylose from soluble sugars). The dominant of these five timescales–the two comparable timescales of solid phase reactive dissolution and particle swelling (Table 2)–determine the overall time required for soluble sugar production from solid carbohydrates, while the enzyme adsorption at the pore scale, being a prerequisite for reaction and swelling, play a crucial role in determining the reducing sugar yields (Table 1, Fig. 6c). Thus, we show that the three pore scale phenomena of enzyme adsorption, solid phase reactive dissolution and particle swelling together determine how long it would take to convert solid hemicelluloses to soluble sugars and how much reducing sugar will be produced.

Further, non-competitive product inhibition in the solid phase significantly increases the reaction time (Table 2b) and reduces sugar yields (Fig. 6b,c). Smart reactor design strategies for transporting the products (xylose and xylobiose) away from the solid carbohydrate particles through diffusion or membrane separation would enhance sugar yields and reduce hydrolysis time, especially when the process is scaled up.

## Conclusions

The crucial first half of the multi-scale hydrolysis of amorphous hemicelluloses is governed by its smallest scale–the pore-scale, where the three phenomena of non-equilibrium adsorption of enzymes, rapid enzyme-catalyzed reactive dissolution from the pore surface, and polymer swelling together determine the soluble sugar yield in the first five hours. An ‘optimal substrate loading’ of 5 mg/ml (above which substrate inhibition sets in) accelerates non-equilibrium enzyme adsorption and solid hemicellulose depolymerization in the pores, resulting in maximum xylose yield (16.6% in 24 h), with the pore-scale phenomena contributing more than three-quarters of the total soluble sugars produced from hemicellulose hydrolysis. Smart reactor design strategies for transporting the products (xylose and xylobiose) away from the solid carbohydrates through diffusion or membrane separation would reduce the non-competitive product inhibition, accelerate hemicellulose hydrolysis and significantly enhance sugar yields, especially when the hydrolysis process is being scaled up for large scale bio-alcohol fermentation.

## Methods

### Materials

Cellulose (Avicel PH101), xylan (beechwood) and endo-(1,4)-*β*-xylanase derived from *Trichoderma longibrachiatum* are purchased from Sigma-Aldrich, USA. Arabinoxylan (wheat) is purchased from Megazyme, and lignocellulose (*Bambusa bambos*) is collected from local area. Sodium acetate anhydrous, glacial acetic acid, 3,5-dinitrosalysilic acid, phenol, potassium sodium tartrate tetrahydrate, and sodium sulfite are purchased from Merck. D-xylose, sodium hydroxide (pellets), benzoic acid, thiourea, potassium bromide, and borax are purchased from SRL, Mumbai, India, and ortho-toluidine is purchased from Loba Chemie, Mumbai, India. All the chemicals used in this study are of analytical grade.

### Substrate characterization

#### Fourier Transform Infrared Spectroscopy (FTIR) analysis

The chemical structure of beechwood xylan is analyzed using FT-IR spectrometer (Spectrum 100, PerkinElmer, USA) at a spectral resolution of 4 cm^−1^ and a scanning range of 400 to 4000 cm^−1^. The KBr disk is made by mixing the dried sample (105 °C for 8 h) with KBr at a ratio of 1:100 w/w and pressing them at 5 Kg/cm^2^ pressure. The peaks are analyzed using Spectrum software.

#### X-ray Diffraction (XRD) analysis

X-ray diffraction spectrum is measured by X’Pert-PRO diffractometer (PANalytical, The Netherlands) using CuK_α_ radiation consisting of K_α1_(λ = 1.5406 Å) and K_α2_ (λ = 1.5444 Å) components. The intensity of the diffracted rays is recorded in the scan range (2θ) of 5–90° with a step size of 0.033° and 19.68s per step.

#### Surface area and pore size measurements

The surface area and the pore size of xylan are measured from the N_2_ adsorption-desorption isotherms obtained at 77 K in a surface area analyzer (Quantachrome, USA), after keeping the sample under high vacuum at 100 °C for 8 h. The specific surface area is calculated by the Brunauer-Emmett-Teller (BET) equation, and the average pore size and the total pore volume are calculated by the Barrett-Joyner-Halenda (BJH) method.

#### Surface morphology

The surface morphologies and the pore structure of xylan, endoxylanase and the solid fractions resulting from the adsorption and the hydrolysis experiments are studied by a Field Emission Scanning Electron Microscope (FESEM) (JSM7610F, JEOL, Japan). The dried samples are mounted on a brass specimen stub with conductive carbon tape, and a sputter coating of 4 nm thickness with platinum is made on each sample under vacuum at 20 mA for 70s using an auto fine coater (JEC3000FC, JEOL). The surface images are captured at various magnifications ranging from 3000 to 100,000 times.

### Adsorption assay

The adsorption of endoxylanse on the solid surface of the hemicellulose (xylan) is measured at various enzyme to substrate ratios (*E*_0_/*C*_*s*_ = 0.1, 0.2, 0.3, and 0.4) with substrate loading varying from 1 to 5 mg/ml in 10 ml of sodium acetate buffer solution (0.1 M, pH 5.0) at 4 °C for 1, 4, 5, and 8 h with frequent mixing. After centrifugation at 11000 rpm and 4 °C for 15 min, the aliquots are collected for estimation of free soluble enzyme concentration. The free endoxylanase activity is determined by measuring the reducing sugar concentration released from the reaction with xylan at 40 °C for 30 min. The amount of adsorbed enzyme is determined by subtracting the free soluble enzyme concentration in the aliquot from the initial concentration of enzyme.

In order to determine the distribution of adsorbed enzymes between pores and external surfaces, the solid xylan particles are soaked in the buffer solution for 5 h prior to the addition of enzyme to the reactor, and the quantification of the enzyme adsorption protocol stated above is followed.

### Enzymatic hydrolysis

The enzymatic hydrolysis is carried out with a mixture of endoxylanase (0.25 mg/ml) and various xylan loadings (1, 2, 3, 5, and 8 mg/ml) in 10 ml total volume of 0.1 M sodium acetate buffer (pH 5.0) at 40 °C for 24 h inside an incubator in an aseptic environment, with intermittent shaking at 150 rpm. Hydrolysates are collected at various hydrolysis times, chilled in an ice-water bath for 2 min, centrifuged at 11000 rpm for 10 min at 4 °C, and assayed for xylose and reducing sugar.

In order to quantify the effect of pore scale on the overall solid phase hemicellulose hydrolysis, the solid substrate with a loading of 5 mg/ml is soaked in the buffer solution for 5 hours prior to the hydrolysis, and the rest of the hydrolysis process as stated above is followed.

### Analytical methods

The concentration of reducing sugar in the hydrolysate is measured by Dinitrosalicylic Acid (DNS) method^43^, and the xylose concentration is measured using o-toluidine-acetic acid reagent^44^,^45^. The supernatant is mixed with the respective reagent in a ratio of 1:1 (v/v) for reducing sugar estimation and 20:1 (μl/ml) for xylose measurement. The sample-reagent mixtures are boiled for 15 min and 9 min at 100 °C in an oil bath (PolyScience, USA) for reducing sugar and xylose concentration measurement, respectively. The absorbance is measured at 540 nm and 480 nm for reducing sugar and xylose estimation, respectively, using UV-visible Spectrophotometer (Cary 100, Agilent, USA), and their respective concentrations are calculated from the calibration curves using D-xylose as a standard.

### Measurement of solid substrate dissolution and polymer swelling by Dynamic Light Scattering

The dissolution of solid substrate particles and the swelling of the polymer in the hydrolyzing medium in the time course of enzymatic hydrolysis of hemicellulose are studied by measuring the average particle size and the particle size distribution by Dynamic Light Scattering (DLS) technique using zetasizer nanoparticle analyzer (Malvern, UK) with laser at a wavelength of 633 nm and at a constant temperature of 25 °C. A sample of 1.5 ml is taken, and the scattering intensity of the laser is measured at 90° angle with the counting time of 80s for each sample. The data collection is performed by DTS (Nano) software.

### Product inhibition and kinetic parameters

The inverse of the reaction rate (time derivative of the substrate concentration in the solid phase) is plotted against the inverse of the solid substrate concentration at various times, in order to determine the type of product inhibition in the solid phase. The kinetic parameter (*K*_*M,s*_) in the solid phase is calculated from the point of intersection of all the best-fit straight lines. The apparent reaction rate (*V*′_*max,s*_) versus time is used to calculate the maximum reaction rate (*V*_*max,s*_) in the solid phase. The xylose inhibition constant (*K*_*s*1_) in the solid phase is estimated from the plot of inverse of reaction rate versus xylose concentration^46^.

### Porosity analysis

The solid residues in the reaction medium are collected at different time intervals after centrifugation and drying at 105 °C for 8 h for the determination of porosity in the yet-unreacted biomass. The porosities in the solid xylan and in the solid residues are calculated using the data obtained from the DLS and the BET analyses.

## Additional Information

**How to cite this article**: Dutta, S. K. and Chakraborty, S. Pore-scale dynamics of enzyme adsorption, swelling and reactive dissolution determine sugar yield in hemicellulose hydrolysis for biofuel production. *Sci. Rep.* **6**, 38173; doi: 10.1038/srep38173 (2016).

**Publisher's note:** Springer Nature remains neutral with regard to jurisdictional claims in published maps and institutional affiliations.

## Supplementary Material

Supplementary Information

## Figures and Tables

**Table 1:** Distribution of the adsorbed enzyme between pores and external surfaces of pre-soaked and control xylan particles at two different adsorption times.

*C*_*s*_ (mg/ml)	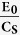	1 h adsorption		5 h adsorption		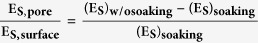	
		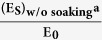	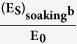	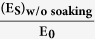	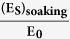	1 h adsorption	5 h adsorption
1	0.1	0.1886	0.0130	0.8856	0.0848	13.53	9.44
	0.2	0.2338	0.0289	0.8790	0.1473	7.08	4.97
	0.3	0.4085	0.0751	0.8785	0.2240	4.44	2.92
	0.4	0.5730	0.1094	0.8809	0.3163	4.24	1.79
2	0.1	0.2271	0.0223	0.9443	0.1207	9.19	6.82
	0.2	0.4985	0.0688	0.9401	0.1959	6.24	3.80
	0.3	0.6120	0.1624	0.9450	0.2799	2.77	2.38
	0.4	0.7469	0.2531	0.9361	0.3605	1.95	1.60
3	0.1	0.3677	0.0440	0.9636	0.1309	7.35	6.36
	0.2	0.6688	0.1114	0.9650	0.2156	5.00	3.48
	0.3	0.7266	0.2075	0.9577	0.2888	2.50	2.32
	0.4	0.8370	0.3083	0.9614	0.3759	1.72	1.56
4	0.1	0.6388	0.0722	0.9734	0.1367	7.85	6.12
	0.2	0.7975	0.1533	0.9761	0.2245	4.20	3.35
	0.3	0.8654	0.2524	0.9703	0.2981	2.43	2.26
	0.4	0.9317	0.3522	0.9692	0.3826	1.65	1.53
5	0.1	0.5924	0.0571	0.9792	0.1391	9.36	6.04
	0.2	0.8212	0.1769	0.9816	0.2277	3.64	3.31
	0.3	0.8641	0.2409	0.9778	0.3039	2.59	2.22
	0.4	0.9126	0.3502	0.9747	0.3861	1.61	1.52

*E*_0_/*C*_*s*_ indicates enzyme to substrate ratio; *E*_s_ represents the adsorbed enzyme concentration; ^a^without soaking in buffer solution; ^b^after 5 h of soaking in the buffer solution prior adsorption. *E*_*s,pore*_/*E*_*s,surface*_ is the ratio of the adsorbed enzyme concentration on the pore surface to that on the external surface.

**Table 2:**
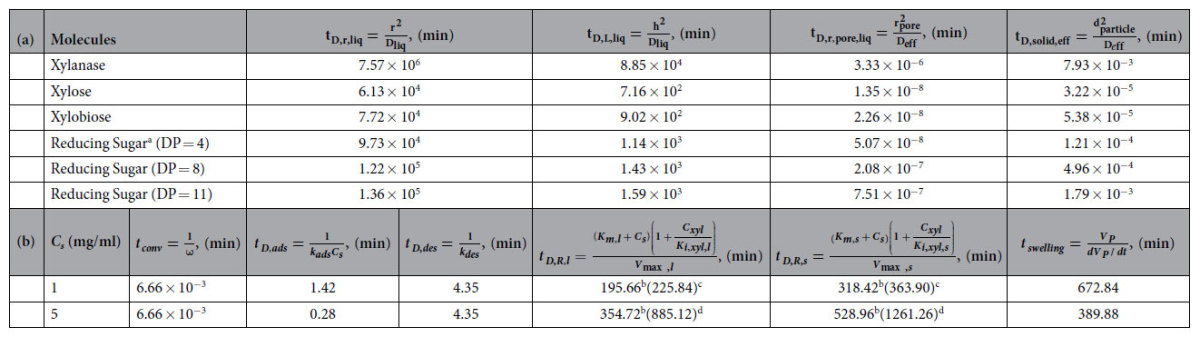
>Transport and reaction timescales of the hemicellulose hydrolysis process.

**a,** Diffusion timescales (in min) of various molecules in hydrolysis. **b,** Convective, adsorption and desorption, reaction and swelling timescales (in min) at two different substrate loadings. In **a,** *t*_*D,r,liq*_ = radial diffusion timescale in the liquid phase; *t*_*D,L,liq*_ = axial diffusion timescale in the liquid phase; *t*_*D,r,pore,liq*_ = radial diffusion timescale in the pores; *t*_*D,solid,eff*_ = longitudinal diffusion timescale in the pores (assuming the maximum pore length to equal the solid particle diameter); *r* = maximum wetted radius of the reactor (3.16 cm); *r*_*pore*_ = average pore radius (4.9 nm); *h* = liquid height in the reactor (6.82 mm); *d*_*particle*_ = average particle diameter (239 nm); *D*_*liq*_ = diffusion coefficient in the liquid phase (cm^2^/sec); *D*_*eff*_ = effective diffusivity in the solid phase (cm^2^/sec). In **b,** *t*_*conv*_ = convective mixing timescale; *t*_*D,ads*_ = adsorption timescale; *t*_*D,des*_ = desorption timescale; *t*_*D,R,l*_ = reaction timescale in the liquid phase; *t*_*D,R,s*_ = reaction timescale in the solid phase; *t*_*swelling*_ = swelling timescale; *ω* = shaking speed (150 rpm); *K*_*i,xyl,l*_ = xylose inhibition constant in liquid phase; *K*_*i,xyl,*s_ = xylose inhibition constant the solid phase; *V*_*p*_ = particle volume.

^a^Diffusion coefficients of reducing sugars calculated in Supplementary Information, section V;

^b^xylose concentration (*C*_*xyl*_) = 0;

^c^*C*_*xyl*_ = 0.0188 mg/ml and

^d^*C*_*xyl*_ = 0.1827 mg/ml at *t* = 5 min.
