# Supplementary material for: Pore-scale dynamics of enzyme adsorption, swelling and reactive dissolution determine sugar yield in hemicellulose hydrolysis for biofuel production
Source: Sci Rep. 2016 Dec 1;6:38173. doi: 10.1038/srep38173 (PMC5131285; doi:10.1038/srep38173)
Supplement: Supplementary Information [file srep38173-s1.pdf]

SUPPLEMENTARY INFORMATION

Pore-scale dynamics of enzyme adsorption, swelling and reactive dissolution determine sugar yield in hemicelluloses hydrolysis for biofuel production

Sajal Kanti Dutta[1] & Saikat Chakraborty[1,2*]

[1]Department of Chemical Engineering, Indian Institute of Technology, Kharagpur 721302, India.

[2]School of Energy Science and Engineering, Indian Institute of Technology, Kharagpur 721302, India.

Table of contents:

I. Surface area and pore characteristics of xylan.

II. FTIR and X-Ray Diffraction (XRD) analyses.

III. Particle volume and porosity dynamics.

IV. Two-phase kinetic model formulation with non-equilibrium and equilibrium adsorption.

V. Diffusion coefficients and effective diffusivities of Reducing Sugars

VI. References

[*]Corresponding author. Tel.: + 91 32222 83930; fax: + 91 32222 82250

E-mail address: saikat@che.iitkgp.ernet.in (Saikat Chakraborty)

I. Surface area and pore characteristics of xylan.

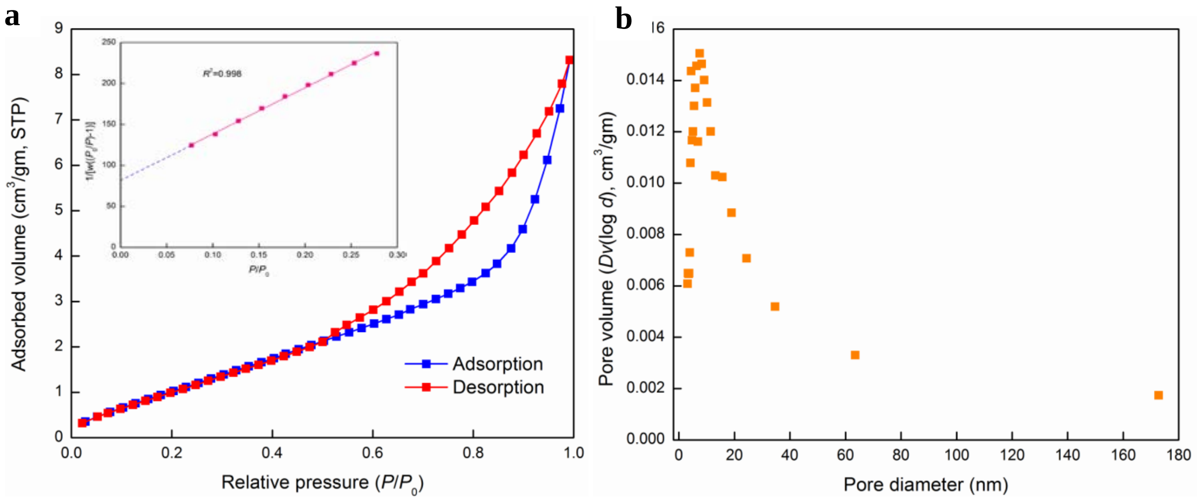

Supplementary Figure S1. a, Nitrogen adsorption-desorption isotherms at 77 K of beechwood xylan. Inset of **a** is the BET plot. **b,** Pore size distribution derived from the desorption branch by the Barrett-Joyer-Halenda (BJH) method. The specific surface area of beechwood xylan from the BET analysis is 5.27 m^2/gm, and the values of the average pore size, the surface area of pores and the total pore volume at a relative pressure of 0.993 from the BJH method are 9.8 nm, 5.837 m^2/gm, and 0.01332 cm^3/gm, respectively. The pore surface area being 1.11 times that of the specific surface area is also indicative of the porous structure of xylan[1].

II. FTIR and X-Ray Diffraction (XRD) analyses.

Fig. 1a in the main text presents the FTIR spectra in transmittance mode, which shows the various vibration bands representing similar functional groups in all four natural polymers, namely, cellulose (Avicel PH101), hemicelluloses (arabinoxylan and beechwoodxylan) and lignocellulose (*Bambusa bambos*). The strong vibration band at 3318 cm^{-1} represents O-H stretching and intermolecular hydrogen bond in phenolic group and in the cellulose structure[2],

the bands at 2924 and 2904 cm^{-1} represent C-H stretching in methyl (CH_3) and methylene (CH_2) groups, the small band at around 1636 cm^{-1} is related to C=C stretching of alkenes (lignin)[3], and H-O-H stretching due to the hydration water present in polysaccharides[4]. A vibration band at 1400 cm^{-1} is related to the C-H bending bond present in the lignin and carbohydrates structures. A band at 1193 cm^{-1} represents C-C and C-O-C stretching vibration bands[5], and the prominent band at 1122 cm^{-1} is attributed to the C-O stretching of different groups. Finally, a small band at around 896 cm^{-1} represents the glycosidic linkages between the sugar units in carbohydrates[6].

Fig. 1b in the main text shows the X-Ray diffraction (XRD) spectra of different biomass samples. The diffracted rays from the cellulose and Bambusa bambos samples produce a constructive interference that satisfy the Bragg equation ($n\lambda=2d\,\sin\theta$, where n is a positive integer, λ is the wavelength of the incident rays, d is the spacing between reflecting planes, and θ is the angle between the planes and the incident rays), while, it does not produce any constructive interference for the hemicellulosic biomasses (xylan, arabinoxylan), resulting in sharp peaks for these two substrates in the diffractogram. The relative amount of crystalline material in each sample is calculated based on the peak height method[8]:

$$CI = \frac{I_{002|2\theta=22.8^o} - I_{am|2\theta=18^o}}{I_{002|2\theta=22.8^o}} \times 100, \qquad \text{(S1)}$$

where CI is the crystallinity index, $I_{002|2\theta=22.8^o}$ represents the maximum intensity of diffraction from the 002 lattice planes at 2θ=22.8°, and $I_{am|2\theta=18^o}$ is the amorphous scattering intensity at 2θ=18°, and the CI values obtained from the experimentally measured I_{002} and I_{am} intensities are 54.2 and 52.2% for cellulose and Bambusa bambos, respectively, while the values of CI for xylan and arabinoxylan are -82.8 and -26.5%, respectively. The negative value of CI, absence of

the constructive interference in the XRD patterns, and the broad scattering peak at 2θ=20° indicate the amorphous structure of xylan and arabinoxylan.

III. Particle volume and porosity dynamics.

The total particle volume of the xylan substrate in the reactor at any time *t* is given by

$$V_s = \frac{\pi d_{p,m}^3 C_s}{6\rho_s \int_{d_{p0,\min}}^{d_{p0,\max}} n_{s0} d_{p0}^2 d(d_{p0})}, \qquad \text{(S2)}$$

$d_{p0,m}$ and $d_{p,m}$ are the average particle sizes at t=0 and at any time t, respectively, of the spherical xylan particles, ρ_s is the density of the solid hemicellulose, $d_{p0,max}$, and $d_{p0,min}$ are the maximum and the minimum particle sizes at t=0, respectively, n_{s0} is the solid particle number fraction of particles size $d_{p0,m}$ in the reactor obtained using DLS, and C_s is the initial substrate loading.

The maximum pore pressure drop (ΔP_{pore}) mentioned in the main text is calculated as 1.966×10^7 N/m^2 (=194 atm), using the formula

$$\Delta P_{pore}=4\sigma Cos\theta/d_{pore}, \qquad \text{(S3)}$$

where d_{pore} is the average pore diameter of xylan (=9.8 nm), and σ and θ are the surface tension (=0.0631 N/m) and the average contact angle (=40.25°), respectively, of the hydrolyzing media, measured by a Goniometer (Fig. 2d).

In Fig. 4b in the main text, the temporal dynamics of the hemicellulose's porosity $\varepsilon_p(t)$ is calculated as

$$\varepsilon_p = 1 - \left(1 - \varepsilon_{p0}\right)\left(\frac{w_t}{w_0}\right)\left(\frac{d_{p0,m}^3}{d_{p,m}^3}\right), \tag{S4}$$

where ε_p and ε_{p0} (=$6V_{pore,0}/(d_{p0,m}S_{s0})$) are the porosities at any time t and at t=0, respectively, S_{s0} and $V_{pore,0}$ are the specific external surface area and the total pore volume of the substrate at t=0, respectively, w_0 and w_t are the weights of the substrate at t=0 and at any time t, respectively. Fig. 4b shows a power law increase in porosity with time.

IV. Two-phase kinetic model formulation with non-equilibrium and equilibrium adsorption.

In the main text, equation (2) represents the adsorption kinetics of the enzyme on the solid hemicellulose, while equations (4) and (5) give the enzyme-catalyzed depolymerizati on kinetics of hemicellulose in the solid and liquid phases, respectively, and equations (6)-(11) represent the non-competitive inhibition kinetics in the solid (m=s) and liquid (m=l) phases.

The rate of change of the adsorbed enzyme concentration in the solid phase is

$$\begin{aligned}\frac{d[E_s]}{dt} &= k_{ads}[E_l]\left(\Omega_{\max}\sum_{i=12}^{n}[X_i]-[E_s]\right)-k_{des}[E_s]-k_s[E_s]\sum_{i=12}^{n}(i-1)[X_i]+\left(k_1+k_s^{-1}\right)\sum_{i=12}^{n}[E_sX_i] \\ &\quad -k_{l1}[E_s][X_1]+k_{l1}^{-1}[E_sX_1]-k_{s2}[E_s][X_2]+k_{s2}^{-1}[E_sX_2].\end{aligned} \tag{S5}$$

For a xylose polymers of chain length i, (i–1) bonds are available for complex formation.The mass balance equations for the intermediate complexes in the solid phase are

$$\frac{d[E_sX_1]}{dt} = k_{s1}[E_s][X_1]-k_{s1}^{-1}[E_sX_1]-k_s[E_sX_1]\sum_{i=12}^{n}(i-1)[X_i]+k_s^{-1}\sum_{i=12}^{n}[E_sX_1X_i], \tag{S6}$$

$$\frac{d[E_sX_2]}{dt} = k_{s2}[E_s][X_2]-k_{s2}^{-1}[E_sX_2]-k_s[E_sX_2]\sum_{i=12}^{n}(i-1)[X_i]+k_s^{-1}\sum_{i=12}^{n}[E_sX_2X_i], \tag{S7}$$

$$\frac{d[E_sX_i]}{dt} = k_s(i-1)[E_s][X_i] - (k_2 + k_s^{-1})[E_sX_i] - k_{s1}[X_1][E_sX_i] + k_{s1}^{-1}[E_sX_1X_i] - k_{s2}[X_2][E_sX_i] + k_{s2}^{-1}[E_sX_2X_i], \quad \text{(S8)}$$

$$\frac{d[E_sX_1X_i]}{dt} = \left\{k_{s1}[X_1][E_sX_i] - k_{s1}^{-1}[E_sX_1X_i]\right\} + \left\{k_s(i-1)[E_sX_1][X_i] - k_s^{-1}[E_sX_1X_i]\right\}, \quad \text{(S9)}$$

$$\frac{d[E_sX_2X_i]}{dt} = \left\{k_{s2}[X_2][E_sX_i] - k_{s2}^{-1}[E_sX_2X_i]\right\} + \left\{k_s(i-1)[E_sX_2][X_i] - k_s^{-1}[E_sX_2X_i]\right\}. \quad \text{(S10)}$$

The mass balance equations for the intermediate complexes in the liquid phase are

$$\frac{d[E_lX_1]}{dt} = k_{l1}[E_l][X_1] - k_{l1}^{-1}[E_lX_1] - k_l[E_lX_1]\sum_{i=3}^{11}(i-1)[X_i] + k_l^{-1}\sum_{i=3}^{11}[E_lX_1X_i], \quad \text{(S11)}$$

$$\frac{d[E_lX_2]}{dt} = k_{l2}[E_l][X_2] - k_{l2}^{-1}[E_lX_2] - k_l[E_lX_2]\sum_{i=3}^{11}(i-1)[X_i] + k_l^{-1}\sum_{i=3}^{11}[E_lX_2X_i], \quad \text{(S12)}$$

$$\frac{d[E_lX_i]}{dt} = k_l(i-1)[E_l][X_i] - (k_2 + k_l^{-1})[E_lX_i] - k_{l1}[X_1][E_lX_i] + k_{l1}^{-1}[E_lX_1X_i] - k_{l2}[X_2][E_lX_i] + k_{l2}^{-1}[E_lX_2X_i], \quad \text{(S13)}$$

$$\frac{d[E_lX_1X_i]}{dt} = \left\{k_{l1}[X_1][E_lX_i] - k_{l1}^{-1}[E_lX_1X_i]\right\} + \left\{k_l(i-1)[E_lX_1][X_i] - k_l^{-1}[E_lX_1X_i]\right\}, \quad \text{(S14)}$$

$$\frac{d[E_lX_2X_i]}{dt} = \left\{k_{s2}[X_2][E_lX_i] - k_{s2}^{-1}[E_lX_2X_i]\right\} + \left\{k_l(i-1)[E_lX_2][X_i] - k_l^{-1}[E_lX_2X_i]\right\}. \quad \text{(S15)}$$

Assuming quasi-steady state for intermediate complexes in the solid and liquid phases allows us to equate equations (S6)-(S8), (S11)-(S13) to zero. Since reactions (6)-(11) (in the main text) attain equilibrium, the terms inside the curly brackets of equations (S9), (S10), (S14) and (S15) equal zero. Rearranging equations (S6)-(S15) gives the following forms, where the subscript *m*=*s* for the solid phase and *m*=*l* for the liquid phase:

$$[E_mX_1] = \frac{[E_m][X_1]}{K_{m1}}, \quad \text{(S16)}$$

$$\left[E_m X_2\right] = \frac{\left[E_m\right]\left[X_2\right]}{K_{m2}}, \tag{S17}$$

$$\left[E_m X_i\right] = \frac{(i-1)\left[E_m\right]\left[X_i\right]}{K_{M,m}}, \tag{S18}$$

$$\left[E_m X_1 X_i\right] = \frac{(i-1)\left[E_m\right]\left[X_1\right]\left[X_i\right]}{K_{M,m} K_{m1}}, \tag{S19}$$

$$\left[E_m X_2 X_i\right] = \frac{(i-1)\left[E_m\right]\left[X_2\right]\left[X_i\right]}{K_{M,m} K_{m2}}, \tag{S20}$$

where $K_{m1} = \frac{k_{m1}^{-1}}{k_{m1}}$, $K_{m2} = \frac{k_{m2}^{-1}}{k_{m2}}$, $K_{M,s} = \frac{k_1 + k_s^{-1}}{k_s}$, $K_{M,l} = \frac{k_2 + k_l^{-1}}{k_l}$.

The rates of the formation of the smaller chain hemicelluloses in the solid phase are given by

$$\begin{aligned} \frac{d\left[X_i\right]}{dt} &= -k_s (i-1)\left[E_s\right]\left[X_i\right] + k_s^{-1}\left[E_s X_i\right] + \left\{-k_s (i-1)\left[E_s X_2\right]\left[X_i\right] + k_s^{-1}\left[E_s X_2 X_i\right]\right\} \\ &\quad + \left\{-k_s (i-1)\left[E_s X_1\right]\left[X_i\right] + k_s^{-1}\left[E_s X_1 X_i\right]\right\} + 2k_1 \sum_{j=i+1}^{n} \frac{\left[E_s X_j\right]}{(j-1)}, \quad i \geq 12. \end{aligned} \tag{S21}$$

The rates of the formation of the products – xylose (X_1), xylobiose (X_2) and other reducing sugars (X_i, $3 \leq i \leq 11$) – in the liquid phase are given by

$$\begin{aligned} \frac{d\left[X_1\right]}{dt} &= \left\{-k_{s1}\left[E_s\right]\left[X_1\right] + k_{s1}^{-1}\left[E_s X_1\right]\right\} + \left\{-k_{s1}\left[X_1\right]\sum_{i=12}^{n}\left[E_s X_i\right] + k_{s1}^{-1}\sum_{i=12}^{n}\left[E_s X_1 X_i\right]\right\} \\ &\quad + \left\{-k_{l1}\left[E_l\right]\left[X_1\right] + k_{l1}^{-1}\left[E_l X_1\right]\right\} + \left\{-k_{l1}\left[X_1\right]\sum_{i=3}^{11}\left[E_l X_i\right] + k_{l1}^{-1}\sum_{i=3}^{11}\left[E_l X_1 X_i\right]\right\} \\ &\quad + 2k_2 \sum_{j=3}^{11} \frac{\left[E_l X_j\right]}{(j-1)} + 2k_1 \sum_{j=12}^{n} \frac{\left[E_s X_j\right]}{(j-1)}, \end{aligned} \tag{S22}$$

$$\begin{aligned} \frac{d\left[X_2\right]}{dt} &= \left\{-k_{s2}\left[E_s\right]\left[X_2\right] + k_{s2}^{-1}\left[E_s X_2\right]\right\} + \left\{-k_{s2}\left[X_2\right]\sum_{i=12}^{n}\left[E_s X_i\right] + k_{s2}^{-1}\sum_{i=12}^{n}\left[E_s X_2 X_i\right]\right\} \\ &\quad + \left\{-k_{l2}\left[E_l\right]\left[X_2\right] + k_{l2}^{-1}\left[E_l X_2\right]\right\} + \left\{-k_{l2}\left[X_2\right]\sum_{i=3}^{11}\left[E_l X_i\right] + k_{l2}^{-1}\sum_{i=3}^{11}\left[E_l X_2 X_i\right]\right\} \\ &\quad + 2k_2 \sum_{j=3}^{11} \frac{\left[E_l X_j\right]}{(j-1)} + 2k_1 \sum_{j=12}^{n} \frac{\left[E_s X_j\right]}{(j-1)}, \end{aligned} \tag{S23}$$

$$\frac{d[X_i]}{dt}=\left\{-k_l(i-1)[E_l][X_i]+k_l^{-1}[E_lX_i]\right\}+\left\{-k_l(i-1)[E_lX_1][X_i]+k_l^{-1}[E_lX_1X_i]\right\}$$
$$+\left\{-k_l(i-1)[E_lX_2][X_i]+k_l^{-1}[E_lX_2X_i]\right\}+2k_2\sum_{j=i+1}^{11}\frac{[E_lX_j]}{(j-1)}+2k_1\sum_{j=12}^{n}\frac{[E_sX_j]}{(j-1)},\ 3\le i\le 11. \qquad \text{(S24)}$$

Endoxylanase randomly cleaves theβ-(1→4)-glycosidic bonds in the hemicelluloses[9] with high probability of bond cleavage for the third and fourth bonds from the reducing end[10]. In the absence of a complete map of the probability of bond cleavage for all bonds in the substrate with DP of 75-250[11] at t=0, an equal probability has been considered for the formation any chain i from a longer chain length j (i<j), which is quantified bythe term 2/(j-1)in equations (S21)-(S24).

The terms inside the curly brackets of the equations (S21)-(S24)) equal zero, and substituting equations (S16)-(S20) in equations (S21)-(S24) gives the rates of sugar formation in the solid (equation (15)) and the liquid (equations (13) and (24)) phases.

Substituting equations (S16)-(S20) in equation (12) (in the main text), the free enzyme concentration in the liquid phase is obtained as

$$[E_l]=\frac{[E_0]-[E_s]\left(1+\frac{[X_1]}{K_{s1}}+\frac{[X_2]}{K_{s2}}\right)\left(1+\frac{1}{K_{M,s}}\sum_{i=12}^{n}(i-1)[X_i]\right)}{\left(1+\frac{[X_1]}{K_{l1}}+\frac{[X_2]}{K_{l2}}\right)\left(1+\frac{1}{K_{M,l}}\sum_{i=3}^{11}(i-1)[X_i]\right)}. \qquad \text{(S25)}$$

For the case of non-equilibrium adsorption of enzyme in the solid phase along with the equilibrium reactions, the rate of change of the adsorbed enzyme concentration follows equation (3) in the main text. For equilibrium adsorption of enzyme on the solid substrate, the enzyme concentration in the solid phase follows the Langmuir adsorption isotherm equation[12] of the following form:

$$\left[E_s^*\right]=\frac{\sigma_{ad}\Omega_{\max}[C_s]\left[E_l^*\right]}{1+\sigma_{ad}\left[E_l^*\right]}, \qquad \text{(S26)}$$

Substitution of equation (S26) in equations (13)-(15) (in the main text), and $[E_l]=[E^*_l]$ give the dynamics of formation of X_i in the solid and the liquid phases at equilibrium, while $[E_s]=0$ gives the rate of formation of xylose and reducing sugars in the liquid phase after 11 h, when the hydrolysis system is assumed to have transitioned to a single phase one.

Number average Degree of Polymerization (DP$_n$): The DP$_n$ of the polymeric hemicellulosic substrate is a measure of the available free glycosidic bonds for enzyme-substrate complex formation in the solid and the liquid phases, and the rate of change of DP$_n$ during the non-equilibrium adsorption of enzyme in the enzymatic hydrolysis process is given by

$$\frac{d}{dt}\left(\frac{1}{DP_n}\right)=\frac{\frac{d}{dt}\sum_{i=1}^{n}[X_i]}{\sum_{i=1}^{n}\left(i[X_i]\right)}=\frac{1}{\sum_{i=1}^{n}\left(i[X_i]\right)}\left([E_l]\left(\frac{k_2}{K_{M,l}}\right)\sum_{i=3}^{11}(i-1)[X_i]+[E_s]\left(\frac{k_1}{K_{M,s}}\right)\sum_{i=12}^{n}(i-1)[X_i]\right). \qquad \text{(S27)}$$

Substituting equation (S26) in equation (S27), we obtain the temporal dynamics of DP$_n$ during equilibrium adsorption (5-11 h), while $[E_s]=0$ gives the rate of change of DP$_n$ in the liquid phase after 11 h of hydrolysis (i.e., when the system has transitioned to the single (liquid) phase).

V. Diffusion coefficients and effective diffusivities of Reducing Sugars.

The relationship between the diffusion coefficient of xylose and its concentration in aqueous solution is given by

$$D_{xyl}=-6.54\times10^{-9}C_{xyl}+7.49\times10^{-6}, \qquad \text{(S28)}$$

where D_{xyl} and C_{xyl} are the diffusion coefficient of xylose (cm^2/sec) and xylose concentration (mg/ml), respectively. Equation (S28) is the best-fit straight line obtained from experimental

results[13] available in the literature, which shows that the diffusion coefficient of xylose decreases linearly with the increase of its concentration.

The diffusion coefficient of a Reducing Sugar molecule of chain length *i* is proportional to its (Molecular Weight)$^{-1/3}$ and is correlated with the xylose's diffusion coefficient in aqueous solution as[14]

$$\frac{D_{RS,i}}{D_{xyl}} = \left(\frac{M_{xyl}}{M_{RS,i}} \right)^{1/3}, \qquad \text{(S29)}$$

where $D_{RS,i}$is the diffusion coefficient of reducing sugar of chain length *i*, and M_{xyl} and $M_{RS,I}$ ($=i\times M_{xyl}$) are the molecular weights of xylose and the Reducing Sugar molecule of chain length *i*, respectively.

The effective diffusivity of the molecule in a porous solid is related to the diffusion coefficient by the following expression[15].

$$D_{eff} = \frac{D_{liq}\varepsilon_p H}{\tau}, \qquad \text{(S30)}$$

Where D_{eff} and D_{liq} are the effective diffusivity and the diffusion coefficient of molecule, respectively, ε_p is the porosity of the solid, τ is the tortuosity factor in the solid (τ=1.6[16]), $H=(1-\Upsilon)^2(1-2.1044\Upsilon+2.089\Upsilon^3-0.948\Upsilon^5)$, and Υ is the ratio of the solute diameter to the pore diameter.The diameters of Reducing Sugar molecules are calculated using the expression: $d_{RS,i}=i\times d_{xyl}$, where d_{xyl} is the diameter of xylose (0.64 nm[17]), while the diameter of xylanase is 3.75 nm[18].

Molecules	D_{liq}, (cm^2/sec)	D_{eff}, (cm^2/sec)
Xylanase[19]	1.78×10^{-7}	1.20×10^{-9}
Xylose	1.08×10^{-5}	2.95×10^{-7}
Xylobiose (DP=2)	8.60×10^{-6}	1.77×10^{-7}
Reducing Sugar (DP = 4)	6.82×10^{-6}	7.89×10^{-8}
Reducing Sugar (DP = 8)	5.42×10^{-6}	1.92×10^{-8}
Reducing Sugar (DP = 11)	4.87×10^{-6}	5.33×10^{-9}

Supplementary Table S1. Diffusion coefficients (D_{liq}) and effective diffusivities (D_{eff}) of enzyme and reducing sugars in enzymatic hydrolysis of hemicellulose (xylan).

VI. References

1. Lu, P. & Hsieh, Y-L. Highly pure amorphous silica nano-disks from rice straw. *Powder Technol.* **225**, 149–155 (2012).
2. Kondo, T. The assignment of IR absorption bands due to free hydroxyl groups in cellulose. *Cellulose* **4**, 281–292 (1997).
3. Poletto, M.P., Zattera, A.J. & Santana, R.M.C. Structural differences between wood species: evidence from chemical composition, FTIR spectroscopy, and thermogravimetric analysis. *J. Appl. Polym. Sci.* **126**, 336–343 (2012).
4. Kacurakova, M., Belton, P.S., Wilson, R.H., Hirsch, J. & Ebringerova, A. Hydration properties of xylan-type structures: an FTIR study of xylooligosaccharides. *J. Sci. Food Agric.* **77**, 38–44 (1998).
5. Xu, F. *et al*. Analysis and characterization of acetylated sugarcane bagasse hemicelluloses. *Int. J. Polym. Anal. Charact.* **9**, 229–244 (2004).
6. Sun, X.F. *et al.* Characteristics of degraded hemicellulosic polymers obtained from steam exploded wheat straw. *Carbohydr. Polym.* **60**, 15–26 (2005).
7. Waser, J. Pictorial representation of the Fourier method of X-ray crystallography. *J. Chem. Educ.* **45**, 446–451 (1968).
8. Segal, L., Creely, J.J., Martin, A.E. Jr. & Conrad, C.M. An empirical method for estimating the degree of crystallinity of native cellulose using the x-ray diffractometer. *Text. Res. J.* **29**, 786–794 (1959).
9. Bastawde, K.B. Xylan structure, microbial xylanases, and their mode of action. *World J. Microbiol. Biotechnol.* **8**, 353–368 (1992).

10. Bray, M.R. & Clarke, A.J. Action pattern of xylo-oligosaccharide hydrolysis by *Schizophyllum commune*xylanase A. *Eur. J. Biochem.* **204**, 191–196 (1992).

11. Timell, T.E. & Syracuse, N.Y. Recent progress in the chemistry of wood hemicelluloses. *Wood Sci. Technol.* **1**, 45–70 (1967).

12. Zhang, Y-H. P. & Lynd, L.R. Toward an aggregated understanding of enzymatic hydrolysis of cellulose: noncomplexedcellulase systems. *Biotechnol. Bioeng.* **88**, 797–824 (2004).

13. Ueadaira, H. &Uedaira, H. Diffusion coefficients of xylose and maltose in aqueous solution. *Bull. Chem. Soc. Jpn.* **42**, 2140–2142 (1969).

14. Sano, Y. & Yamamoto, S. Mutual diffusion coefficient of aqueous sugar solutions. *J. Chem. Eng. Jpn.* **26**, 633–636 (1993).

15. Blanch, H.W. & Clark, D.S. *Biochemical Engineering* (Marcel Dekker, New York, 1997).

16. Jacobson, A.J. & Banerjee, S. in *Materials, Chemicals, and Energy from Forest Biomass* (ed. Argyropoulos, D.S.) 219–228 (ACS symposium Series, 2007).

17. Qi, B., Luo, J., Chen, X., Hang, X. & Wan, Y. Separation of furfural from monosaccharides by nanofiltration. *Bioresour. Technol.* **102**, 7111–7118 (2011).

18. Wyman, C.E. *et al*. in *Polysaccharides: Structural Diversity and Functional Versatility* (ed. Dumitriu, S.) 995–1033 (Marcel Dekker, New York, 2005).

19. Whitmire, D. & Maiti, B. Xylanase effects on pulp delignification. *Chem. Eng. Comm.* **189,** 608–622 (2002).
